# CuCr_2_O_4_@rGO Nanocomposites as High-Performance Cathode Catalyst for Rechargeable Lithium–Oxygen Batteries

**DOI:** 10.1007/s40820-017-0175-z

**Published:** 2017-12-08

**Authors:** Jiandi Liu, Yanyan Zhao, Xin Li, Chunge Wang, Yaping Zeng, Guanghui Yue, Qiang Chen

**Affiliations:** 1 Department of Materials Science and Engineering, College of Materials, Xiamen University, Xiamen, 361005 People’s Republic of China; 2 Fujian Provincial Key Laboratory of Plasma and Magnetic Resonance, Department of Electronic Science, Xiamen University, Xiamen, 361005 People’s Republic of China

**Keywords:** CuCr_2_O_4_@rGO nanocomposites, Cathode catalyst, Lithium–oxygen batteries

## Abstract

Rechargeable lithium–oxygen batteries have been considered as a promising energy storage technology because of their ultra-high theoretical energy densities which are comparable to gasoline. In order to improve the electrochemical properties of lithium–oxygen batteries (LOBs), especially the cycling performance, a high-efficiency cathode catalyst is the most important component. Hence, we aim to demonstrate that CuCr_2_O_4_@rGO (CCO@rGO) nanocomposites, which are synthesized using a facile hydrothermal method and followed by a series of calcination processes, are an effective cathode catalyst. The obtained CCO@rGO nanocomposites which served as the cathode catalyst of the LOBs exhibited an outstanding cycling performance for over 100 cycles with a fixed capacity of 1000 mAh g^−1^ at a current density of 200 mA g^−1^. The enhanced properties were attributed to the synergistic effect between the high catalytic efficiency of the spinel-structured CCO nanoparticles, the high specific surface area, and high conductivity of the rGO.

**Electronic supplementary material:**

The online version of this article (10.1007/s40820-017-0175-z) contains supplementary material, which is available to authorized users.

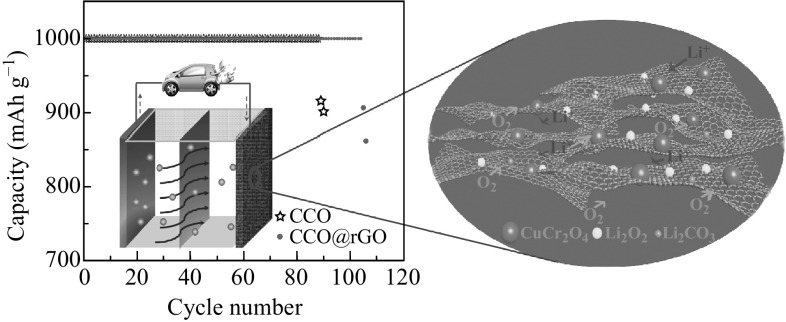

## Highlights

- CuCr_2_O_4_@rGO nanocomposites were facilely synthesized using the hydrothermal method.
- The CuCr_2_O_4_@rGO nanocomposites demonstrated an outstanding cycling performance for over 100 cycles with a fixed capacity of 1000 mAh g^−1^ at a current density of 200 mA g^−1^.

## Introduction

In the past decades, the deterioration of global climate and the growing shortage of oil resources have become major issues for mankind. Therefore, the need to develop new kinds of power sources that are environmental-friendly and sustainable in terms of development is becoming increasingly urgent. Nowadays, several renewable energies, such as solar radiation, wind, waves, and geothermal energy, have been expected to solve these major issues. However, all of these sources exhibit the same demerit of intermittence which restricts their practical applications [1–4]. More recently, Li-ion and other battery-related technologies have been the most convenient form of energy storage and are the key solution to the global energy crisis. In particular, lithium–oxygen batteries (LOBs) have received unprecedented attention owing to their superhigh theoretical energy density (1–2 kWh kg^−1^) [5], which is comparable to gasoline and far exceeds any other subsistent rechargeable electrochemical energy storage technology [6–8]. However, there are still many challenges that need to be addressed concerning practical development [7–9]. The first and most critical problem is the high discharge/charge overpotential. It is well known that Li_2_O_2_ is generated in the course of discharge at about 2.7 V and then decomposed at a high voltage up to 4 or 4.5 V in the process because of its poor conductivity [10]. During the cycling processes, the deterioration of the electrolyte and the formation of the secondary product Li_2_CO_3_ can occur between the interfaces of the oxygen electrode. The insoluble Li_2_CO_3_ can isolate the Li^+^ and O_2_; this can extremely affect the reversibility and sustainability of the LOBs [11]. In addition, the round-trip efficiency will be reduced directly by the high overpotential. In other words, more energy is wasted during the “input” and “output” conversion process of the batteries.

Therefore, in order to achieve the practical application of LOBs, choosing a reasonable solution to reduce the overpotential of the cycling processes is the most critical step. Among the various available technical solutions, selecting an efficient solid catalyst to reduce the overpotential is a sensible and practical strategy. Various kinds of noble metals (such as Pd, Pt, Au, and Pt–Ir) have been applied as cathode catalysts for rechargeable LOBs, and the cycle stability of the LOBs has been remarkably improved [12, 13]. However, the implication of high costs makes noble metals impractical for LOBs. In this regard, metal oxides with various nanostructures have been widely studied as close substitutes [14–21]. Compared to carbon, the metallic Magnéli-phase Ti_4_O_7_ cathode can greatly reduce the problem of overpotential [14]. Some perovskite-based structure materials have also been reported as a high-performance cathode catalyst for rechargeable LOBs [22, 23]. Flower-like NiOs with a highly hierarchical porous structure have been synthesized and used as a cathode material for LOBs which resulted in an outstanding cycling performance of over 80 cycles at a current density of 200 mA g^−1^ [21]. Yuan et al. studied the SnO_2_-based cathode catalyst for lithium–air batteries [24]. Due to the advantageous synergistic effect of bimetals, CuCo_2_O_4_ nanoparticles exhibited excellent catalytic activity for LOBs [25]. Recently, using a capacity-controlled method (1000 mAh g^−1^) at a current density of 100 mA g^−1^, mesoporous Cr_2_O_3_ nanotubes applied as a cathode catalyst for LOBs demonstrated excellent cyclic stability up to 50 cycles [16]. Ru-decorated Co_3_O_4_ nanosheets grown on carbon textiles offered high capacity, improved round-trip efficiency, and enhanced cycling capability [26]. The Co_3_O_4_@MnO_2_/Ni nanocomposite exhibited a small discharge/charge voltage gap of about 0.76 V and a stable cycle life [27]. With a cutoff capacity of 600 mAh g^−1^ at 400 mA g^−1^, the Li–O_2_ cell with MnCo_2_O_4_/KB catalyst can be cycled for more than 70 cycles [28]. These studies indicate that the round-trip efficiency and cycling capability can be effectively enhanced by the synergistic effect between the multicomponent and hierarchical structure. Moreover, Dai et al. reported the preparation of low-cost, efficient, metal-free, binder-free, and hierarchically porous air electrodes with nitrogen-doped graphene that showed good catalytic performance [29, 30].

Copper chromate (CuCr_2_O_4_, CCO), as one of the promising family member of spinel structure mixed transition metal oxides, has been widely studied in photocatalytic water-splitting for hydrogen production, pollution control, and solid propellants [31–35]. In our previous work, we found that CCO is an active anode material for lithium-ion batteries, which means that it can function as an efficient electrode catalyst for LOBs [36]. Thus, in this study, we synthesized the CCO and CuCr_2_O_4_@rGO (CCO@rGO) and investigated their electrochemical properties as the cathode catalysts of LOBs.

## Experimental

### Synthetization of Materials

All reagents used were AR grade and without further purification. First, 8 mmol CuCl_2_·2H_2_O and 16 mmol CrCl_3_·6H_2_O were added in 30 mL deionized water. The pH of the solution was then set to 8 by adding NH_4_OH dropwise. Meanwhile, 6.4 mmol cetyltrimethyl ammonium chloride (CTAC) and 1.2 mmol hydrazine monohydrate (80% aqueous solution) were mixed with 6 mL deionized water to form sol, and the two solutions were combined and stirred for 30 min. Subsequently, the mixture was sealed in a 50-mL Teflon (polytetrafluoroethylene)-lined stainless-steel autoclave and heated to 180 °C for 24 h. After the autoclave naturally cooled down to room temperature, the precursor was collected after being washed with deionized water several times and then dried at 60 °C in an oven for 12 h. To obtain the final product, the resulting green powder was calcined at 650 °C for 6 h in air in an electric furnace with the ramp at 1 °C min^−1^ [37]. For comparison, the conditions for adding only CuCl_2_ or CrCl_3_ were also studied.

The modified Hummer’s method was used to prepare the graphene oxide (GO) solution (see supporting information). In a typical experiment, the CCO powders previously obtained were mixed with the GO aqueous suspensions and subjected to ultrasonication for 1 h to achieve stable mixed suspensions. Following this step, the mixed suspensions were transferred into a Teflon vessel and heated to 180 °C for 3 h. After cooling down, the solutions were washed with deionized water several times and then dried at 60 °C in an oven for 12 h to obtain the final products [38].

### Characterizations

The crystal structures of the samples were characterized using X-ray diffraction (XRD, Bruker D8 with Cu *K*α 40 kV, 40 mA, Germany). The morphologies of the products were determined using scanning electron microscopy (SEM, SU-70, Japan) and LEO-1530 (Germany)) and transmission electron microscopy (TEM, JEM 2100, 200 kV, Japan). The thermogravimetric analysis (TGA) measurement was taken using an SDT-Q600 thermal analyzer (USA) at 10 °C min^−1^ under atmospheric conditions. Raman spectroscopy was carried out in order to examine the graphene sheets using a LabRAM HR UV/Vis/NIR PL by Horiba Jobin–Yvon of France. Fourier transform infrared spectroscopy (FT-IR) was applied to detect the functional groups of GO, RGO, and CCO with a NICOLET IS10 device (Thermo Fisher Scientific, USA).

### Electrochemical Measurements

The Li–O_2_ batteries (CR2032) were assembled in an Ar-filled glove box (< 0.1 ppm of H_2_O and 0.1 ppm of O_2_). The slurry was prepared by mixing the active materials, Ketjen carbon, and polyvinylidene difluoride (PVDF) in a weight ratio of 80:10:10. The slurry was pasted on a carbon paper and dried at 80 °C for 12 h in a vacuum oven. The mass loading of active materials/KB/PVDF was about 1.0–1.3 mg cm^−2^. Lithium metal was chosen as the counter electrode, and a Whitman glass microfiber separator filled with electrolyte was used as a separator. The electrolyte was 1 M LITFSI (lithium bis-(trifluoromethanesulfonyl)-imide) in TEGDME (tetraethylene glycol dimethyl ether). The quantity of electrolytes for each cell was 150 μL. The assembled Li–O_2_ battery was stored in a box filled with pure O_2_ (99.999%) when tested. Electrochemical impedance spectroscopy investigations were conducted on an Autolab 1.9 electrochemistry workstation. The NEWARE battery test system was used to test the galvanostatic discharge/charge capacities at different current densities in the potential range of 2.0–4.5 V.

## Results and Discussion

During the fabrication process, a suitable pH value for the precursor solution could be achieved by using NH_4_OH, which maintained the solution under alkaline condition. Thus, the Cu and Cr ions could quickly form a Cu–Cr hydroxide precipitation. Meanwhile, the effective addition of the surfactant CTAC allowed the solution to reach the critical micelle concentration (CMC) and provided Cu and Cr ions with positive charges and the head of the micelle region with a negative charge, thus forming sphere-like nanoparticles in the process [39, 40]. Moreover, the addition of hydrazine is another crucial step to prevent the Ostwald ripening process by a reasonable degree [41]. After the calcination processes in air, the Cu–Cr precursor transformed into the spinel structure CuCr_2_O_4_ nanoparticles via an oxidation reaction process.

**Fig. 1:**
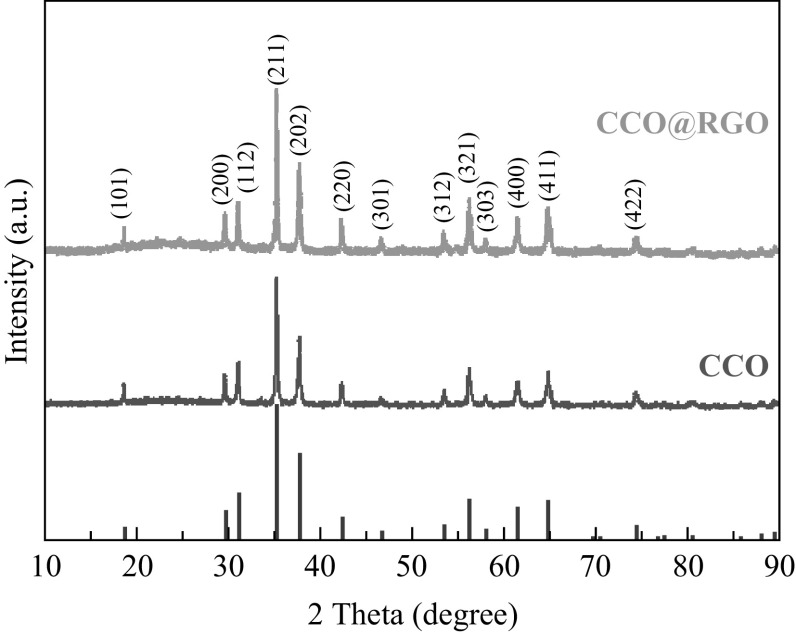
XRD patterns of CCO and CCO@rGO

XRD was used to examine the phase structure and purity of the obtained products. The final products which were well crystallized after the calcination processes were applied on the amorphous structure of the synthesized precursors (Fig. S1). As shown in Fig. 1, all the diffraction peaks can be well indexed to crystal planes of the spinel structure CuCr_2_O_4_ phase (JCPDS card No. 88-0110). In addition, the XRD results of the synthesized products by adding only CuCl_2_ and CrCl_3_ show that the resulting products were well crystallized CuO and Cr_2_O_3_ (Fig. S1). From the TGA result (Fig. S2), it is obvious that the weight loss of the precursor was nearly stopped completely when the temperature was over 600 °C, which indicates that the precursor was totally transformed into CCO after calcinations [42].

To achieve combination of the CCO nanoparticles and rGO nanosheets, the hydrothermal method was selected. The hydrothermal process can significantly remove the functional groups and reduce GO to rGO. Furthermore, during the hydrothermal process, the rGO was successfully wrapped onto the surface of the CCO nanoparticles under high pressure and temperature. The phase structure of the final product is presented in Fig. 1, which reveals that the CCO nanostructure remained unchanged during the hydrothermal process when rGO was assembled on the surface of the CCO nanoparticles. All the diffraction peaks were indexed to the pure Fd3m-CuCr_2_O_4_ phase structure and conformed to the JCPDS card (No. 88-0110). Moreover, no obvious diffraction peaks of GO or carbon were detected, which indicate that GO was completely converted into rGO after heat treatment at 180 °C for 3 h. The FT-IR spectra of CCO versus CCO@RGO and GO versus RGO are displayed in Fig. S3a, b [37, 43]. It can be observed that the FT-IR spectra are very similar to the CCO@rGO nanocomposite and CCO nanoparticles. In Fig. S3a, the Cr_2_O_4_^2−^ group was detected with two absorption bands at 608 and 517 cm^−1^, respectively, which is compliant with the special spinel structure. The stretching vibrations of the Cr–O–Cu and the Cr–O bonds of the spinel structure (CuCr_2_O_4_) were measured within two absorption bands at about 517 and 620 cm^−1^, respectively. The band presented at 517 and 500 cm^−1^ could be flagged with an asymmetric line broadening, which corresponds to the feature bands of Cu–O. In addition, the representative band of Cr–O was detected at about 557 cm^−1^. The Au and 2Bu modes can be marked with three absorption peaks which were located at about 601, 508, and 432 cm^−1^, respectively. Here, the Cu–O stretching along the [$\overline{1}$01] direction is measured to the peak which was found at 601 cm^−1^, but was observed at about 508 cm^−1^ for the Cu–O stretching along the [101] direction. Besides, no GO functional group was detected after the hydrothermal process, which indicates that GO was totally converted into rGO during the heat treatment process (Fig. S3b), and the result corresponds with those of XRD. The pure rGO Raman spectrum is presented in Fig. S4, in which the disordered (D band) and graphitic (G band) bands were detected at about 1350 and 1590 cm^−1^, respectively. Compared to the traditional rGO synthesis method, in this study, with an efficient and environmental-friendly hydrothermal process, GO was completely reduced to rGO, which was then assembled on the CCO surface directly without any further experimentation. In addition, the data obtained from the nitrogen adsorption/desorption isotherm show that the BET specific surface area of CCO and CCO@rGO nanocomposites was calculated to be 60.1 and 174.8 m^2^ g^−1^, respectively (see Fig. S5). Compared with CCO nanoparticles, the high specific surface area of the CCO@rGO nanocomposites can provide more catalytic activity sites; thus, the electrochemical performance of the LOB was evidently enhanced.

**Fig. 2:**
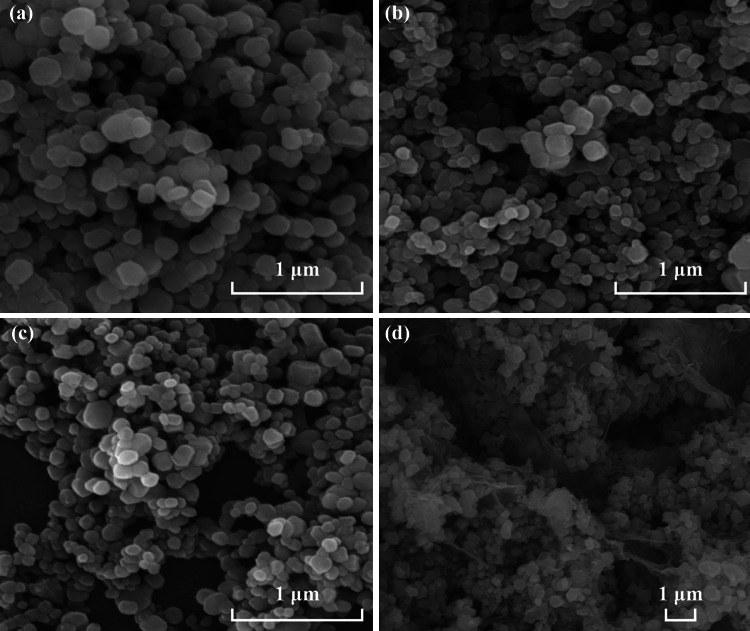
SEM images with different magnifications of **a**, **c** CCO nanoparticles, **b**, **d** CCO@rGO nanocomposites

**Fig. 3:**
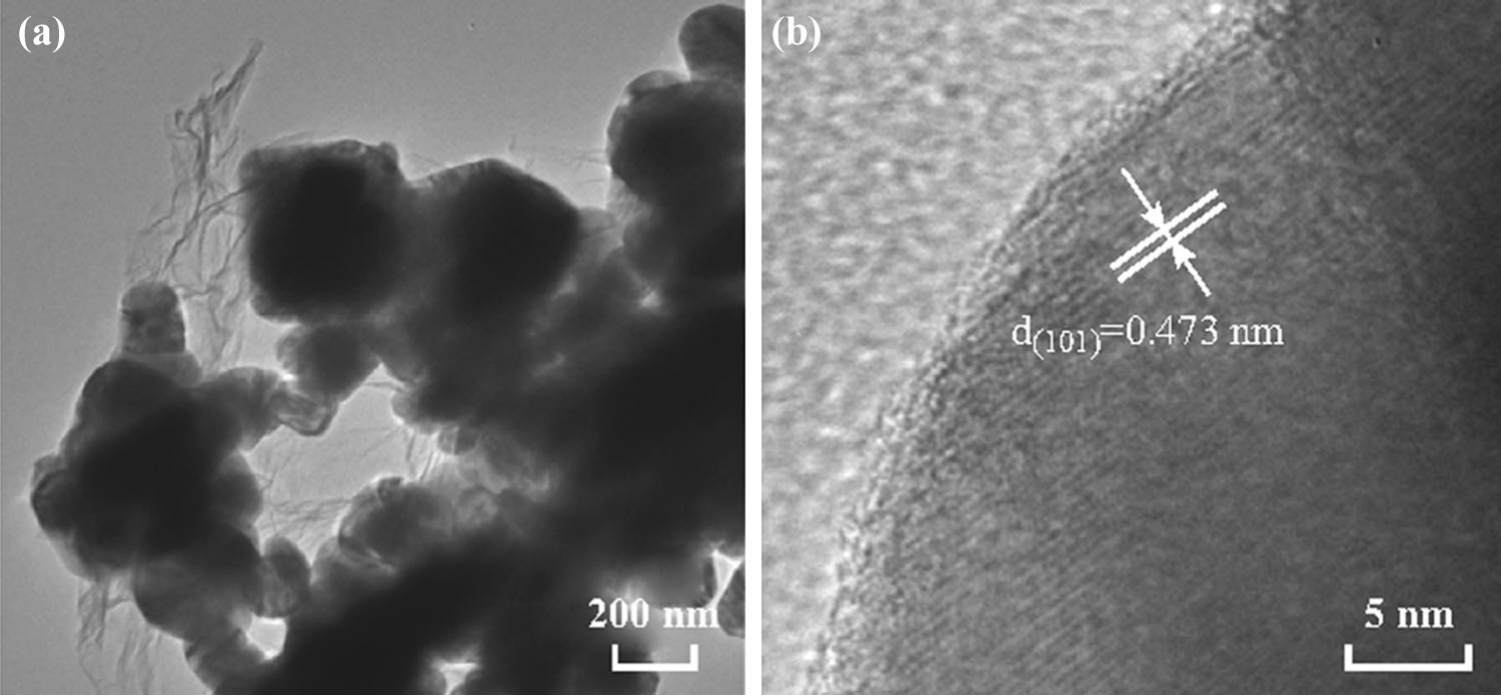
TEM image of **a** CCO@rGO and **b** with corresponding HRTEM pattern

The morphology of the final powder product is shown in Fig. 2. It was found that in the CCO@rGO nanocomposite, the CCO nanoparticles still maintained their morphology and size as bare CCO nanoparticles. The wrapping of the CCO nanoparticles in rGO was also confirmed by the TEM image. As shown in Fig. 3a, rGO was assembled on the surface of the CCO nanoparticles and wrapped to form the nanocomposite. The content of rGO is very lightweight and formed several layers of thin films on the surface of CCO. But with a large superficial area and rapid electron propagation velocity properties, rGO greatly helps in improving the electrical conductivity of the CCO@rGO nanocomposite [29, 38]. A clear lattice fringe with a distance of about 0.473 nm is shown in the HRTEM image of Fig. 3b, which corresponds to the (101) crystal faces of the CCO nanoparticles. TGA was used to calculate the content of rGO in the CCO@rGO nanocomposites, and the final products of CCO@rGO were processed in air from room temperature to 800 °C. Compared with other traditional modification processes, the content of rGO was calculated to be about 8.92% (Fig. S2b). However, it is widely known that a small amount of rGO can greatly improve the properties [29, 37] and also enhance the cycling stability of Li–O_2_ batteries. The diameter distribution of CCO is shown in Fig. S1c, d.

**Fig. 4:**
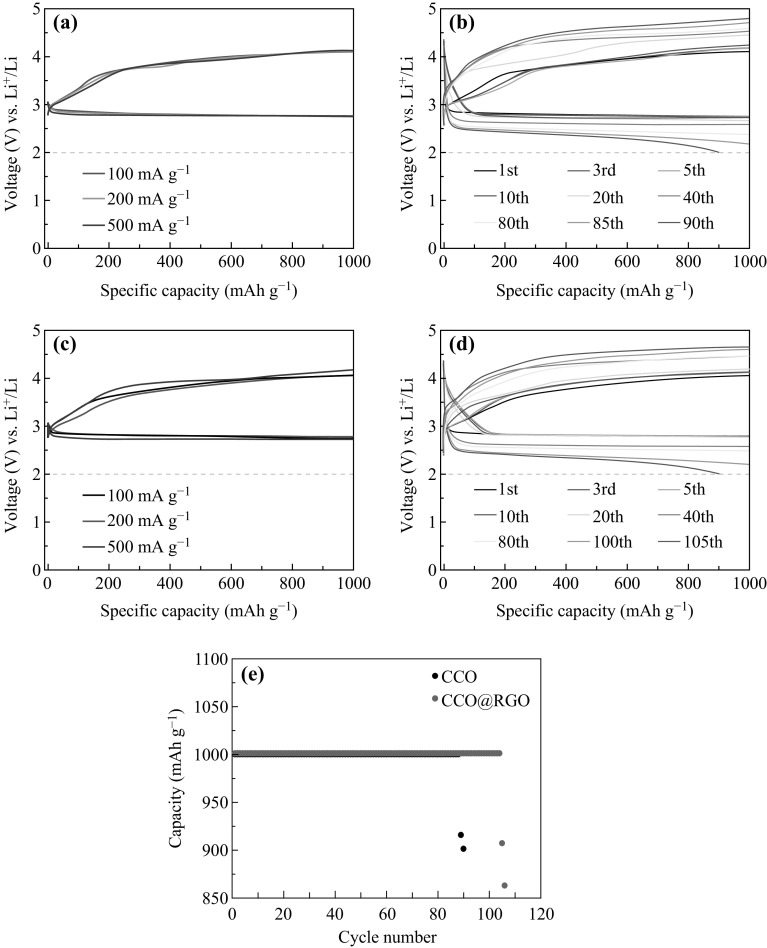
**a**, **c** Galvanostatic discharge and charge curves of the CCO and CCO@rGO cathodes at different current densities (100, 200, and 500 mA g^−1^). **b**, **d** Discharge/charge curves of the CCO@rGO and CCO cathodes. **e** Cyclic stability of the LOBs with CCO@rGO and CCO cathodes. All the discharge and charge processes are measured via a limited capacity of 1000 mAh g^−1^

The CCO and CCO@rGO nanocomposites were employed as cathode catalysts in rechargeable LOBs. Figure 4a, c presents the discharge/charge curves of the LOBs tested using CCO and CCO@rGO cathodes at current densities of 100, 200, and 500 mA g^−1^ for the first cycle. From these figures, the potential plateaus of the discharge process are almost identical and reached a stable level at 2.75 V even when the current densities were extremely different, thus signifying an outstanding ORR catalytic activity of the CCO nanoparticles and CCO@rGO nanocomposites. During the discharge/charge process as shown in Fig. 4a, the overpotential of the different current rate is approximately about 1.1 V. However, the results of CCO@rGO were different. It was found (from Fig. 4c) that the overpotential decreased slightly, and the reduced range was about 0.1 eV at low current densities of 100 and 200 mA g^−1^, but no obvious reduction was observed at a high current density of 500 mA g^−1^. This can be attributed to the superhigh surface area and the excellent electronic transmission capacity of rGO [1, 29, 35]. The discharge/charge curves of CCO@rGO with a limited capacity of 1000 mAh g^−1^ at current densities of 100 and 500 mA g^−1^ are shown in Fig. S6e, f, respectively.

Figure 4b, d shows the discharge/charge curves of the LOBs tested using CCO and CCO@rGO cathodes at a current density of 200 mA g^−1^ for the different cycles. In Fig. 4b, d, both types of cathode show slightly large discharge/charge terminal voltages before 80 cycles. However, when cycling over 85 cycles, the charge terminal voltage of the LOBs assembled using the CCO cathode reached the initial fixed cutoff voltage of 2.0 V. Meanwhile, LOBs assembled using the CCO@rGO cathode cycles more than 100 cycles.

Figure 4e shows the cycle performance of the two cathode types at a current density of 200 mA g^−1^ with a limited capacity of 1000 mAh g^−1^. A remarkable cycling life is observed for 85 and 100 cycles in the LOBs based on CCO and CCO@rGO, respectively. The electrochemical performances of CuO, Cr_2_O_3_, KB, and rGO synthesized using the same method were also measured (Fig. S6). The pure KB electrode provided the poorest electrochemical performance because of its low ORR and OER catalytic activity. Benefiting from the spinel structure of CCO nanoparticles and the synergistic effect from the bimetallic of Cu and Cr, the cycle stability and cycle life were certainly enhanced [17, 24, 32]. Owing to the superhigh surface area and the excellent electronic transmission ability of rGO, the cycleability was significantly enhanced further [30]. The kinetics of ORR and OER for CuCr_2_O_4_@rGO and CuCr_2_O_4_ cathodes in non-aqueous electrolyte was investigated using cyclic voltammetry (CV) in a three-electrode system and within a pure oxygen atmosphere (see Fig. S7b). The results indicate that the CuCr_2_O_4_@rGO cathode exhibits a lower potential ORR initialization but a larger ORR peak current density than CuCr_2_O_4_.

For comparison, various spinel structure mixed transition metal oxide composites used as cathode catalysts for LOBs are listed in Table 1. For example, the as-synthesized needle-like NiCo_2_O_4_ coated on graphene foam delivered a stability of over 80 cycles at a current density of about 400 mA g^−1^ with a limited capacity of 1000 mAh g^−1^ [44]. The Co_3_O_4_ nanofibers fitted on non-oxidized graphene nanoflakes were reported with more than 80 cycles at a current density of about 200 mA g^−1^ with a limited capacity of 1000 mAh g^−1^ [45]. With a limited capacity of 1000 mAh g^−1^, a higher current density stability of the rechargeable LOBs with a current density of about 500 mA g^−1^ was obtained using the CNT@RuO_2_ composite cathode catalysts [46]. Recently, ruthenium oxide-coated ordered mesoporous carbon nanofiber arrays which were synthesized as a catalyst for LOBs presented a very long cycle stability of over 300 cycles at a current density of about 250 mA g^−1^ with limited capacity of 1000 mAh g^−1^. This high-performance cathode catalyst which belongs to the family of the rare ruthenium metal has been investigated by other researchers [47].

**Table 1:** Electrochemical properties of various spinel structure mixed transition metal oxides composites used as the cathode catalysts of LOBs with a limited capacity of 1000 mAh g^−1^

Materials	Morphology	Overpotential (V)	Current density (mA g^−1^)	Cycles number	References
NiCo_2_O_4_@graphene foam	Needle-like NiCo_2_O_4_	0.87	400	At least 80	[44]
Co_3_O_4_/graphene nanoflakes	Co_3_O_4_ nanofibers	–	200	80	[45]
CNT@RuO_2_ composites	RuO_2_ nanoparticles	0.97	500	Over 100	[46]
NiCo_2_O_4_–nitrogen-doped graphene oxide	Flower-like NiCo_2_O_4_	1.76	200	50	[48]
CuCr_2_O_4_@rGO composites	CuCr_2_O_4_ particles	0.99	200	100	Our work
RuO_2_-coated ordered mesoporous carbon nanofiber arrays	–	0.75	250	300	[49]

**Fig. 5:**
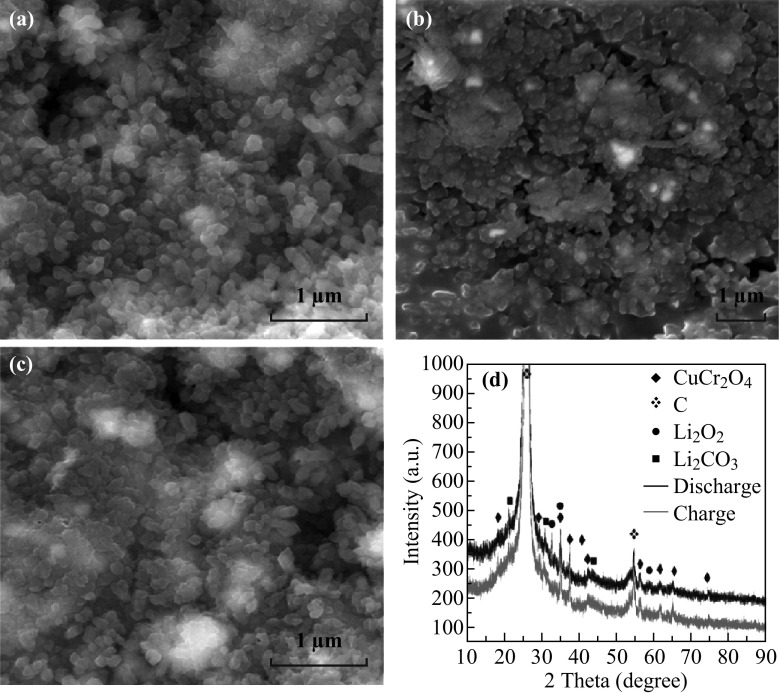
SEM images of CCO@rGO electrodes **a** initial, **b** after discharge, and **c** after recharge stage. **d** XRD patterns of the cathodes after the discharge and recharge stages

For further investigation of the discharge/charge processes of LOBs using CCO@rGO catalysts, a number of batteries were dissembled and then characterized using SEM under different conditions (Fig. 5a–c). As shown in Fig. 5, compared to the original electrode, the surface of the discharged electrode was fully covered by Li_2_O_2_ film. However, after the charge process, the discharge products were decomposed and nearly recovered the morphology of the original state. Figure 5d presents the XRD patterns of the cathodes after the discharge and charge stages. The presence of the peaks of Li_2_O_2_ could be easily identified after discharge, which had initially disappeared after the charge process. This means that Li_2_O_2_ was the main product during the charge/discharge process. It should be noted that Li_2_CO_3_, which was the by-product of the cycle process, was also present [23]. In addition, the electrochemical impedance spectra were recorded and are shown in Fig. S7a. When the LOBs are discharged, the polarization of the electrode is largely increased because of the formation of Li_2_O_2_ with poor conductivity [9, 16]. After the recharge, the polarization is reduced and shows little change compared to the original, which is consistent with the results of the SEM images.

**Fig. 6:**
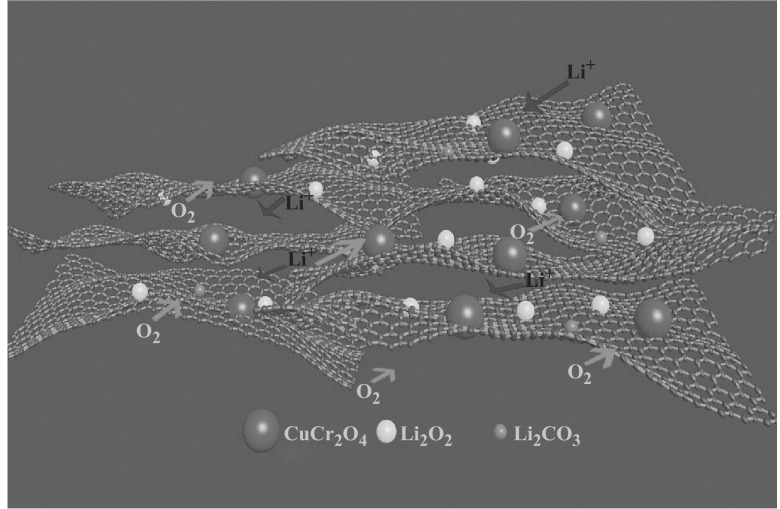
Schematic illustration for the function of LOBs using CCO@rGO catalyst

As shown in Fig. 6, the reasons for the enhanced properties using CCO@rGO catalyst were speculated to be due to the combination of CCO and rGO. The coated rGO with large specific surface area increased the connection between the CCO particles, thus providing a special pathway for Li-ion transport. The combination also improved the entire conductivity of the materials and helped to establish a balance between the conductivity and active sites. Furthermore, the important mechanical property of rGO increases the tolerance of the volume change caused by the formation and decomposition of the discharge product Li_2_O_2_ [29, 30, 38]. Ultimately, because of the advantage of the synergistic effect between CCO nanoparticles and rGO, the electrochemical properties of the LOBs were significantly improved.

The practical mechanism and the specific process can be stated as follows: in the case of pure O_2_, oxygen can be easily absorbed using the superhigh specific surface area of the CCO@rGO nanocomposites [48]. The absorbed O_2_ reacts with Li^+^ which reaches the catalyst at the channel created through the interaction with rGO nanosheets to form LiO_2_. The formation of LiO_2_ would increase the response and synergy with the oxygen-containing species, which were absorbed using CCO nanoparticles in order to produce the Li_2_O_2_ films. Following the charge process, the CCO nanoparticles showed superior catalytic properties, leading to the easy decomposition of Li_2_O_2_. Meanwhile, the graphene substrate, which was curled on the CCO surface, acted as a better electrical conductor for providing rapid transmission [47, 49–51]. With the good catalytic performance of CCO nanoparticles and the great electronic transmission capacity of rGO nanosheets, the lithium peroxide films were decomposed at a lower potential charge of 3.56 V.

## Conclusions

In summary, an effective cathode catalyst material—CuCr_2_O_4_ nanospheres wrapped by reduced graphene oxide nanocomposites, was synthesized using a facile sol–gel method and followed by hydrothermal and calcinations processes. The rGO nanosheet with high specific surface area increased the effective interaction among the electrolyte, catalyst, and oxygen. The improved conductivity also accelerated the Li-ion and electron path speed. In conjunction with the excellent catalytic activity of the spinel structure CCO, the LOBs exhibited a long-term cycling performance of over 100 cycles. In conclusion, the spinel structure transition metal oxide coupled with rGO nanocomposites presented in this paper is believed to be capable of making significant contributions to the future development of LOBs.

## Electronic supplementary material

Below is the link to the electronic supplementary material.

Supplementary material 1 (PDF 1293 kb)
