# Supplementary material for: CuCr2O4@rGO Nanocomposites as High-Performance Cathode Catalyst for Rechargeable Lithium–Oxygen Batteries
Source: Nanomicro Lett. 2017 Dec 8;10(2):22. doi: 10.1007/s40820-017-0175-z (PMC6199065; doi:10.1007/s40820-017-0175-z)
Supplement: Supplementary file 1 — Supplementary material 1 (PDF 1293 kb) [file 40820_2017_175_MOESM1_ESM.pdf]

Supporting Information for

$CuCr_2O_4$@rGO Nanocomposites as High-Performance Cathode Catalyst for Rechargeable Lithium-Oxygen Batteries

Jiandi Liu[1], Yanyan Zhao[1], Xin Li[1], Chunge Wang[1], Yaping Zeng[1], Guanghui Yue[1,*], Qiang Chen[2]

[1] Department of Materials Science and Engineering, College of materials, Xiamen University, Xiamen 361005, People's Republic of China

[2] Fujian Provincial Key Laboratory of Plasma and Magnetic Resonance, Department of Electronic Science, Xiamen University, Xiamen 361005, People's Republic of China

*Corresponding author. E-mail: yuegh@xmu.edu.cn

1 Synthesize the Graphene Oxide (GO) Hydrate Suspension

In a typical synthesized process, 1.2 g graphite was spread into a beaker with a mixture solution which containing 144 mL cold concentrated H_2SO_4 and 16 mL H_3PO_4. Then 7.2 g $KMnO_4$ was added into the above mixture slowly, and the mixture were stirred for 30 min and cooled with ice-bath to keep the temperature as low as possible. Then, the mixture was transferred into water bath with 50 °C for 12 h. Following, cooled with the pure ice cooling system, 3% H_2O_2 were dropped in the suspension until the color of the mixture changes to bright yellow. Finally, the suspension was collected and washed by centrifugation with large amounts of DI water and dried for 24 h in the vacuum. Figure S8 shows the XRD pattern of GO nanosheets. In our experiment, the GO nanosheets were prepared to form the GO solution (2 mg mL^{-1}) by ultrasonic exfoliation.

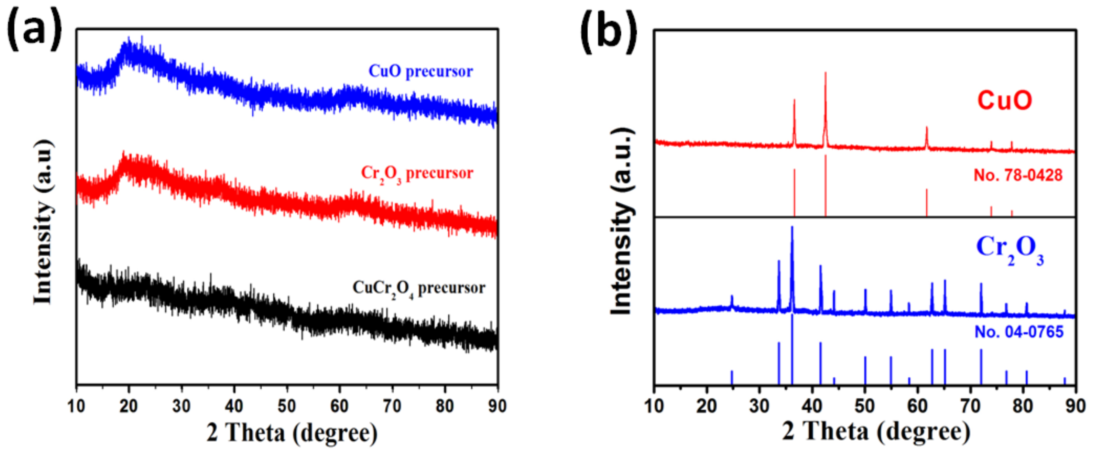

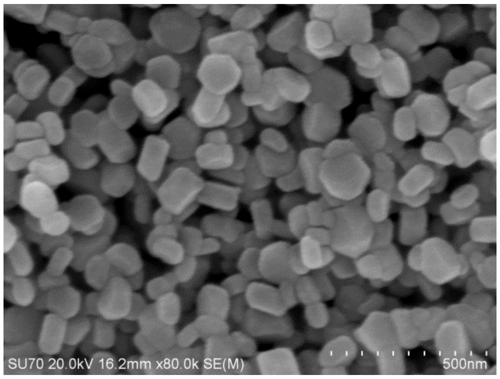

(c)

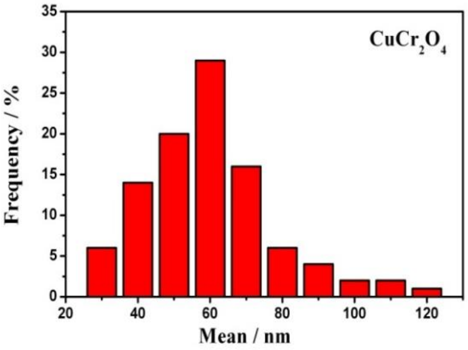

(d)

Fig. S1 XRD patterns of **a** precursors and **b** synthesized CuO and Cr_2O_3. **c** SEM image and **d** diameter distribution of CCO

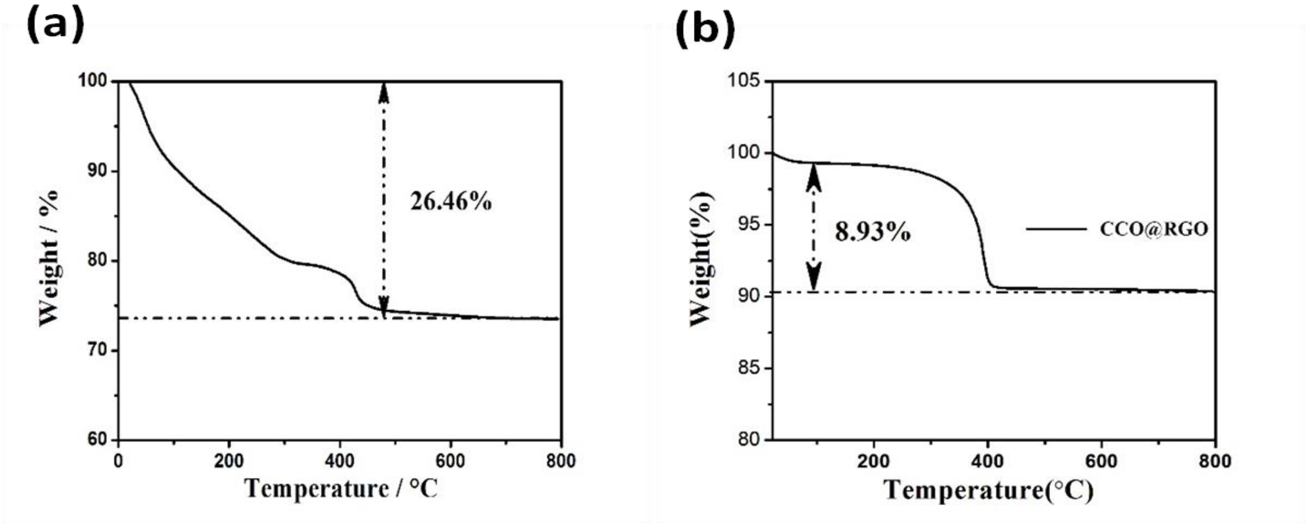

Fig. S2 TGA curves of **a** precursor and **b** CCO@RGO with a heating rates of 10 °C min^{-1}

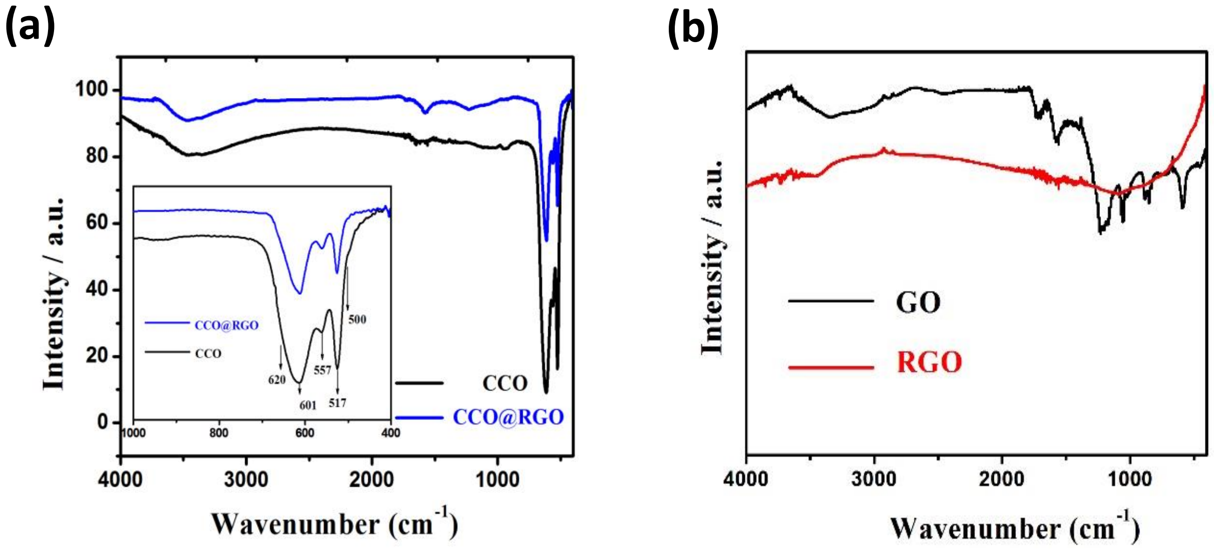

Fig. S3 FTIR spectra of **a** CCO and CCO@rGO, **b** GO and rGO

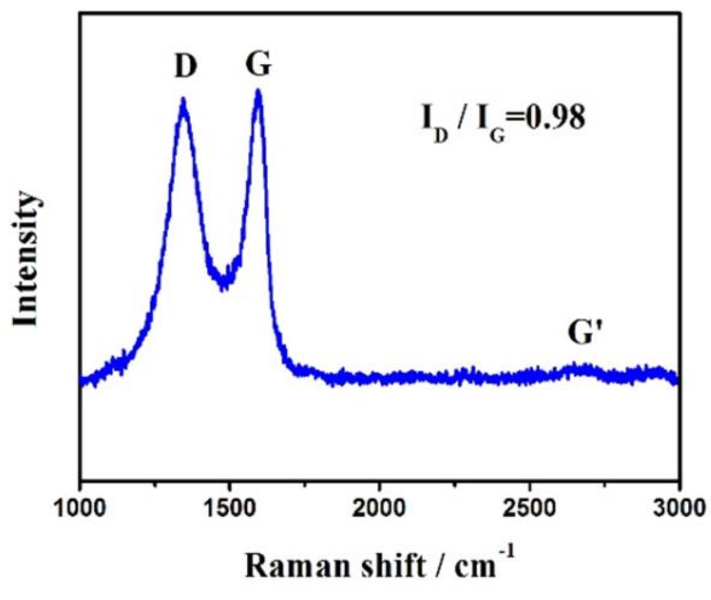

Fig. S4 Raman spectra of RGO reduced by the same method

(a)

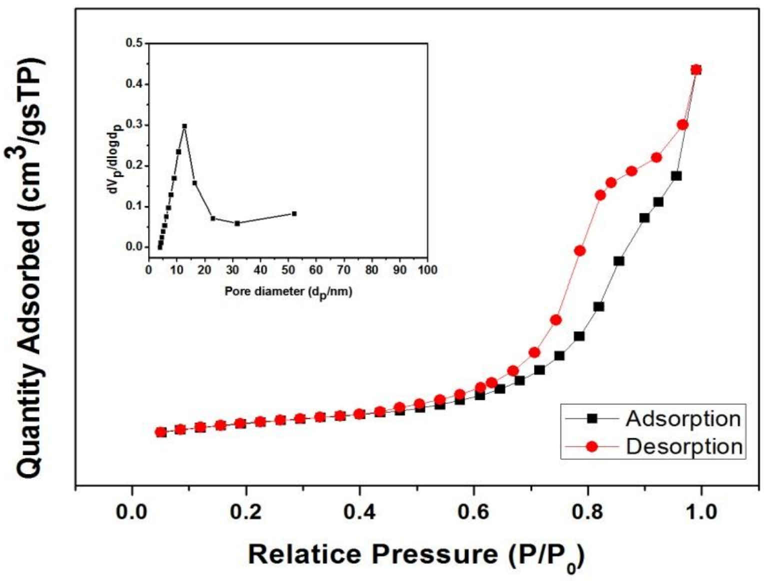

(b)

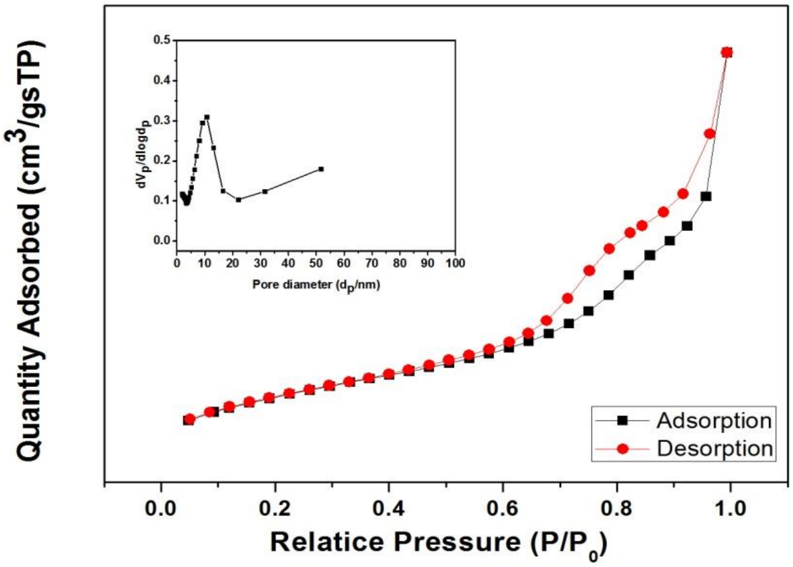

Fig. S5 Nitrogen adsorption−desorption and pore size distribution (inset) curves of **a** CCO and **b** CCO@rGO

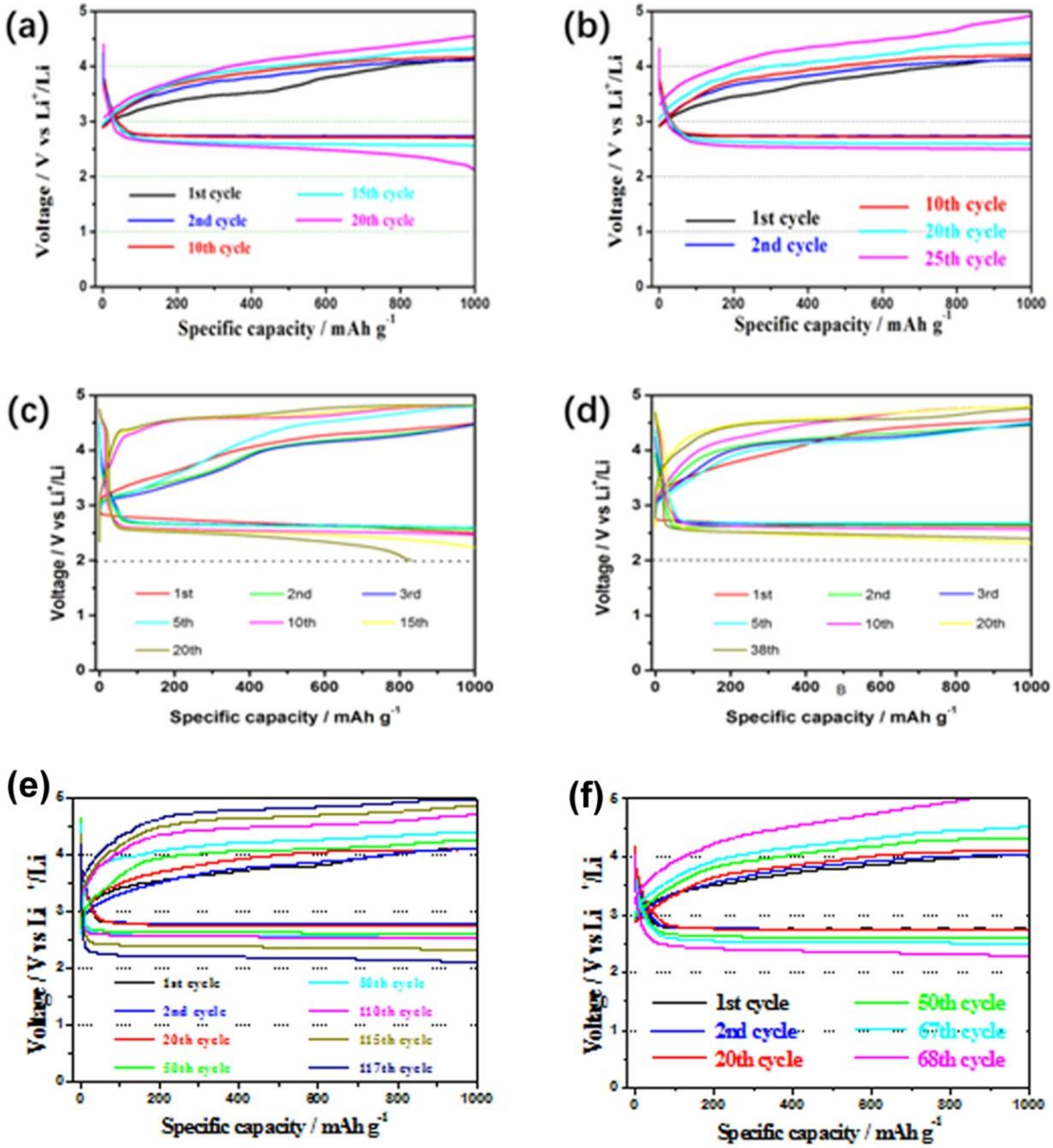

Fig. S6 The discharge/charge curves of **a** CuO, **b** Cr_2O_3, **c** KB and **d** rGO with a limited capacity of 1000 mAh g^{-1} at the current density of 200 mA g^{-1}. **e, f** The discharge/charge curves of CCO@rGO with a limited capacity of 1000 mAh g^{-1} at the current density of 100 and 500 mA g^{-1}

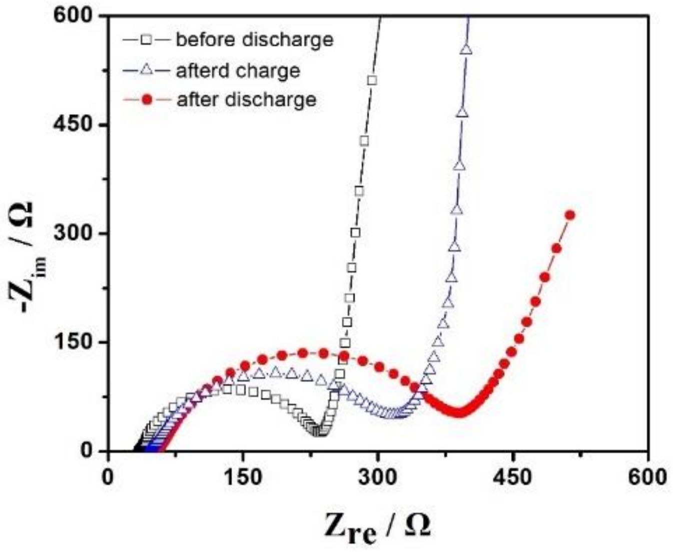

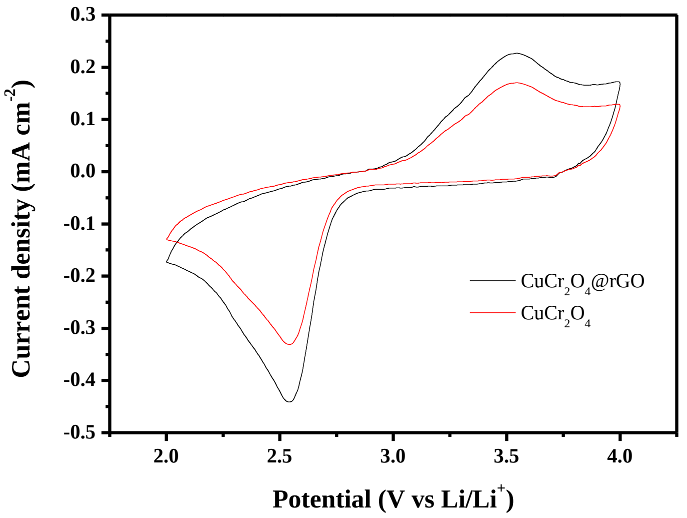

Fig. S7 a EIS results of the cathodes of initial, after discharge, after recharge stage. **b** CV curves of $CuCr_2O_4$ and $CuCr_2O_4$@rGO in 0.1 M LITFSI/DMSO solution at sweep rate of 10 mV s^{-1}

The kinetics of ORR and OER for $CuCr_2O_4$@rGO and $CuCr_2O_4$ cathodes in non-aqueous electrolyte is investigated by cyclic voltammetry (CV) in three electrode system in the pure oxygen atmosphere. One reduction peak at about 2.54 V can be clearly observed, which can be ascribed to the oxygen reduction. The $CuCr_2O_4$@rGO cathode exhibits a lower onset ORR potential and larger ORR peak current density than $CuCr_2O_4$. This indicated that the fast kinetics towards ORR of the $CuCr_2O_4$@rGO nanocomposites. Consistent with the result of the discharge-charge profle, a oxidation peaks at around 3.55 V can be found in the CV curve.

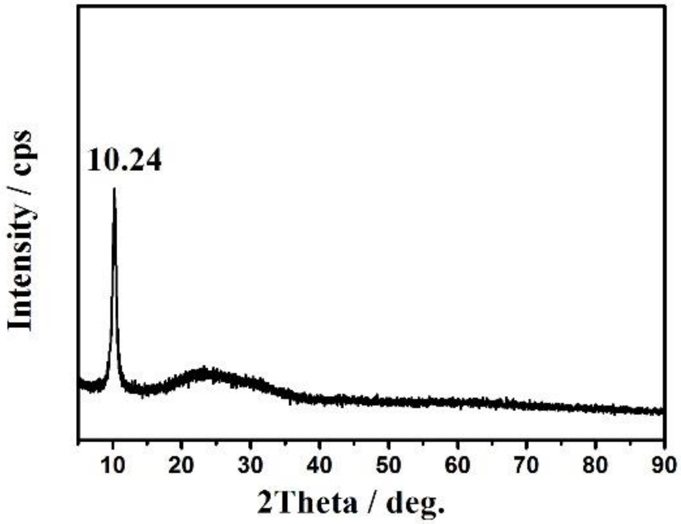

Fig. S8 the XRD pattern of GO prepared by a modified Hummer's method
